# Surgical margin status outcome of intraoperative indocyanine green fluorescence-guided laparoscopic hepatectomy in liver malignancy: a systematic review and meta-analysis

**DOI:** 10.1186/s12893-024-02469-1

**Published:** 2024-06-12

**Authors:** Vorapatu Tangsirapat, Malika Kengsakul, Suwasin Udomkarnjananun, Paiboon Sookpotarom, Mati Rattanasakalwong, Jantaluck Nuchanatanon, Panutchaya Kongon, Kitti Wongta

**Affiliations:** 1 Department of Surgery, Panyananthaphikkhu Chonprathan Medical Center, Srinakharinwirot University, Nonthaburi, 11120 Thailand; 2 Department of Obstetrics and Gynecology, Panyananthaphikkhu Chonprathan Medical Center, Srinakharinwirot University, Nonthaburi, 11120 Thailand; 3 Division of Nephrology, Department of Medicine, Faculty of Medicine, King Chulalongkorn Memorial Hospital, Chulalongkorn University, Bangkok, 10330 Thailand; 4 Department of Surgery, Panyananthaphikkhu Chonprathan Medical Center, 222 Tiwanon Road, Pak Kret, Nonthaburi, 11120 Thailand

**Keywords:** Fluorescence imaging, Hepatectomy, ICG, Indocyanine green, Laparoscopy, Liver neoplasms

## Abstract

**Background:**

Hepatectomy stands as a curative management for liver cancer. The critical factor for minimizing recurrence rate and enhancing overall survival of liver malignancy is to attain a negative margin hepatic resection. Recently, Indocyanine green (ICG) fluorescence imaging has been proven implemental in aiding laparoscopic liver resection, enabling real-time tumor identification and precise liver segmentation. The purpose of this study is to conduct a systematic review and meta-analysis to ascertain whether ICG-guided laparoscopic hepatectomy yields a higher incidence of complete tumor eradication (R0) resections.

**Methods:**

The search encompassed databases such as PubMed, Cochrane Library database, Scopus, ScienceDirect, and Ovid in April 2024, in strict adherence to the Preferred Reporting Items for Systematic Reviews and Meta-Analyses (PRISMA) guidelines. Studies involving patients with malignant liver lesions who underwent ICG-guided laparoscopic hepatectomy and reported R0 resection outcomes were eligible for inclusion in this review.

**Results:**

In a total of seven studies, involving 598 patients, were included in the meta-analysis. The ICG demonstrated a significantly elevated R0 resection rate compared to the non-ICG group [98.6% (359/364) vs. 93.1% (339/364), odds ratio (OR) = 3.76, 95% confidence intervals (CI) 1.45–9.51, *P* = 0.005]. Notably, no heterogeneity was observed (*I*^*2*^ = 0%, *P* = 0.5). However, the subtype analysis focusing on hepatocellular carcinoma [98.2% (165/168) vs. 93.6% (161/172), OR = 3.34, 95% CI 0.94–11.91, *P* = 0.06) and the evaluation of margin distance (4.96 ± 2.41 vs. 2.79 ± 1.92 millimeters, weighted mean difference = 1.26, 95% CI -1.8-4.32, *P* = 0.42) revealed no apparent differences. Additionally, the incidence of overall postoperative complications was comparable between both groups, 27.6% (66/239) in the ICG group and 25.4% (75/295) in the non-ICG group (OR = 0.96, 95% CI 0.53–1.76, *P* = 0.9). No disparities were identified in operative time, intraoperative blood loss, postoperative blood transfusion, and length of hospital stay after the surgery.

**Conclusions:**

The implementation of ICG-guided laparoscopic hepatectomy can be undertaken with confidence, as it does not compromise either intraoperative or postoperative events. Furthermore, the ICG-guided approach is beneficial to achieving a complete eradication of the tumor during hepatic resection.

**Trial registration:**

PROSPERO registration number CRD42023446440.

**Supplementary Information:**

The online version contains supplementary material available at 10.1186/s12893-024-02469-1.

## Background

A malignant tumor of the liver encompasses both primary and metastatic tumors. Liver resection stands as a curative approach for this group of patients [1, 2]. Introduced in 1991, laparoscopic liver resection (LLR) primarily involved wedge liver resection or minor hepatectomies [3]. Subsequently, the prevalence of laparoscopic hepatectomies rapidly increased after the first International Consensus Conference on Laparoscopic Liver Resection (ICCLLR) in 2008 [4]. Nowadays, advancements in surgical techniques and instruments have led LLR into all procedures of hepatectomy including wedge resection, segmentectomy, sectionectomy, and hemihepatectomy [5]. Notably, LLR has been demonstrated to be safe even for hepatopancreatobiliary surgeons with limited experience [6].

While laparoscopic hepatectomy is recognized for its substantial reduction in complications and hospitalization duration, its impact on oncological outcomes remains a subject of debate. The key to diminishing recurrence rates and elevating survival rates lies in achieving complete tumor eradication, or what is referred to as R0 resection [7–10]. The challenge in LLR arises from the inability to manually assess the tumor and being unable to place the ultrasound probe during the transection. So, we cannot see the tumor in real-time during the procedure and might leave it behind. Additionally, intraoperative ultrasound (IOUS) may fail to detect minute superficial tumors [1]. Therefore, novel techniques have been determined to achieve the negative margin.

Indocyanine green (ICG) binds to plasma protein, generating a fluorescent signal. The near-infrared wavelength will create green imaging and subsequently excreted through the biliary tract and liver [11]. ICG has found applications in various procedures, including assessing liver function with the Indocyanine green retention ratio at 15 min (ICGR15) [12], bile duct imaging [13], sentinel lymph node detection in breast cancer [14], aiding in cerebral aneurysm surgery [15], conducting retinal angiography [16], and assessing blood supply perfusion in cardiovascular disease and colorectal surgery [17, 18]. In 2008, Aoki first introduced complications-free fluorescence-guided hepatic resection [19]. The use of ICG guidance in laparoscopic hepatectomy can assist with tumor detection and facilitate real-time liver segmentation during surgery. Our study is designed to determine whether the ICG-guided laparoscopic hepatectomy yields a superior rate of complete tumor resection in cases of hepatic cancer.

## Materials and methods

### Data sources and searches

#### Search strategy

The literature searches were developed in PubMed, Cochrane Library database, Scopus, ScienceDirect, and Ovid from inception to April 2024. The search included the terms “indocyanine green”, “ICG”, indocyanine green fluorescence”, “fluorescence”, “laparoscopy”, “hepatectomy”, “liver cancer”, and “liver neoplasms”. The search was limited to excluding conference papers and articles only involving animals. No authors or subject experts were contacted, and we did not browse unindexed journals in the field. The methods in this review are described based on the Preferred Reporting Items for Systematic Reviews and Meta-Analyses (PRISMA) Checklist [20], the Prisma-S extension to the PRISMA Statement for Reporting Literature Searches in Systematic Reviews [21], and meta-analysis of observational studies (MOOSE) checklist [22]. The study protocol was registered in PROSPERO with the registration number CRD42023446440.

#### Study selection

Studies published in English with adequate information, in accordance with the Strengthening the Reporting of Observational Studies in Epidemiology (STROBE) statement [23], were included in the review. Two authors (VT and KW) independently screened the titles and abstracts of the retrieved electronic citations. Then, they retrieved and reviewed the full texts of the seemingly relevant articles. Any disagreements between VT and KW were resolved through discussion and arbitration by a third author (MK). The reference lists of retrieved articles were searched for potentially missing relevant studies. We included retrospective and prospective cohort studies as well as clinical trials that evaluated no residual tumor (R0) in patients with malignant liver lesions who underwent laparoscopic hepatectomy. Studies that did not report R0 rate or studies published as conference abstracts, case reports, case series, narrative reviews, editorials, letters, and short communications were excluded.

#### Data extraction and quality assessment

The primary outcome of the study is the R0 resection rate, defined as no residual microscopic and macroscopic tumor. Study characteristics were extracted as follows: name of the first author, year of publication, country, study center, sample size, and study design. Patient-related characteristics extracted were mean age, tumor type, intraoperative outcome, and postoperative complications. Minor postoperative complications are defined as Clavien-Dindo classification I-II and major postoperative complications are defined as Clavien-Dindo classification ≥ IIIA. Additional file Table S1 provides The Newcastle-Ottawa Quality Assessment Scale (NOS), which was used to evaluate the quality and the risk of bias in the observational studies included in our meta-analysis.

#### Data synthesis and analysis

Results were synthesized quantitatively by performing random-effects meta-analyses to compute weighted mean difference (WMD) for continuous variables and pooled odds ratios (ORs) for binary variables. All pooled estimations are displayed with 95% confidence intervals (CI). The mean and standard deviation were calculated based on the method described by Wan et al., if not provided in the study [24]. Heterogeneity in study effect sizes was examined using the *I*^*2*^ index and the Q-test *P* value. An *I*^2^ index > 75% indicates medium to high heterogeneity. Categorical variables are presented as numbers (%) and continuous variables as mean ± standard deviation (SD). Statistical significance was defined as a P-value < 0.05. Publication bias was formally assessed using the Egger test. The analyses were performed using Review Manager (RevMan) version 5.4.1 (the Cochrane Collaboration, 2020).

## Results

### Study selection, characteristics, and quality of included studies

**Fig. 1:**
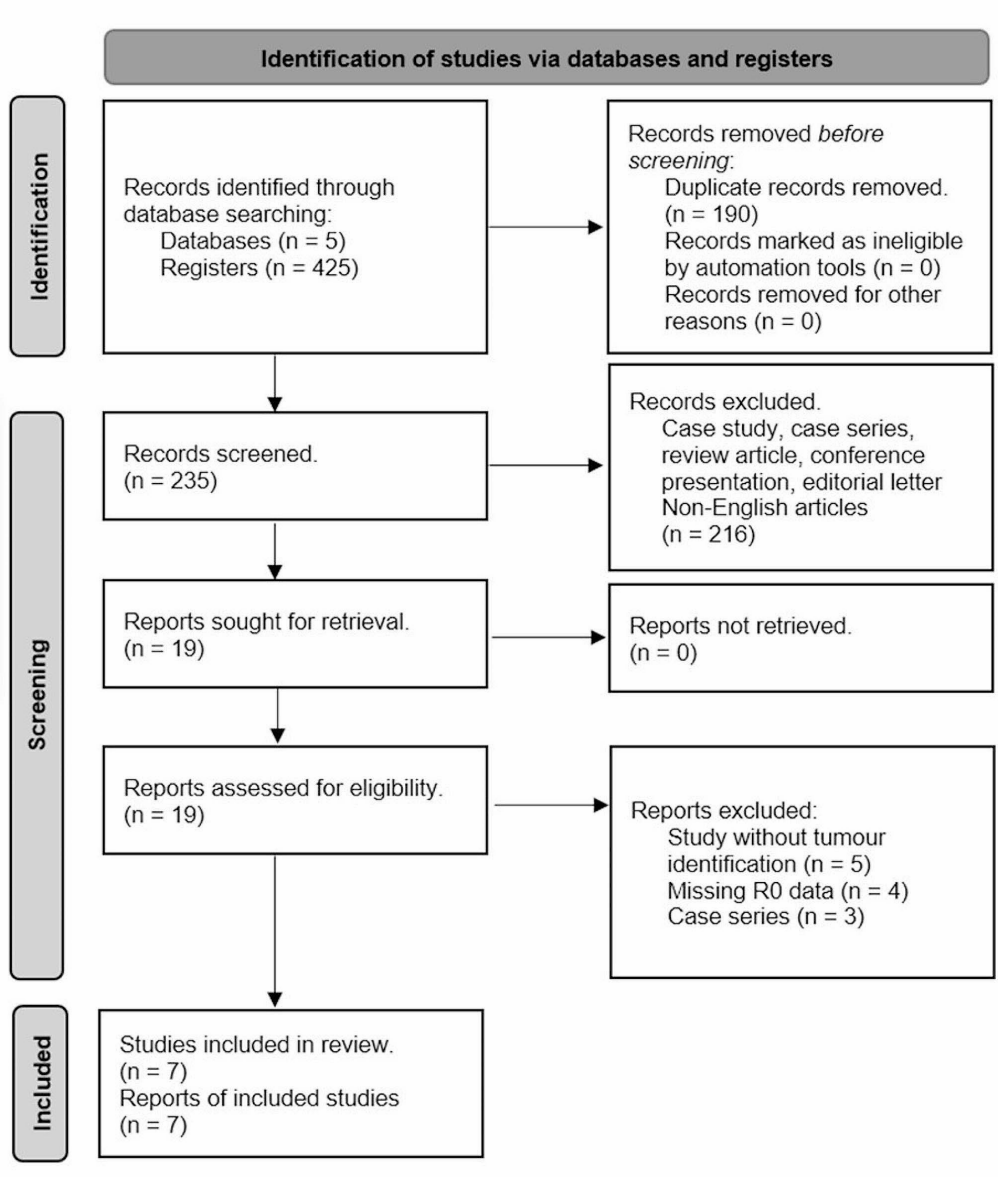
PRISMA flow diagram of the literature selection [20]

A total of 425 abstracts were generated through the initial search, with 190 studies being eliminated due to duplication. Following a thorough review of the abstracts, 228 were subsequently excluded based on the criteria explained in Fig. 1. Conclusively, seven retrospective cohort articles encompassing 598 patients with 327 patients in the non-ICG group, and 271 patients in the ICG group were analyzed [25–31]. The number of tumors was 728 in total, comprising 364 tumors in the non-ICG group, and 364 tumors in the ICG group. The characteristics of the included studies are shown in Table 1. These studies spanned publication dates from 2018 to 2023. In terms of quality assessment, the NOS score exhibited a range from 7 to 9, indicative of good quality.

**Table 1:** Summary of the included studies

References	Year	Country	Sample size			Age		Disease type	ICG application	NOS
			Total	Non-ICG	ICG	Non-ICG	ICG			
Aoki T. et al. [25]	2018	Japan	97	72	25	69 (35–86)	63 (34–84)	HCC/CRLM	Tumor location	9
Wang G. et al. [26]	2022	China	25	11	14	56(45–70)	54.5(46–60)	NETs liver metastases	Tumor location	8
Zhou Y. et al. [27]	2019	China	42	21	21	NA	NA	HCC	Tumor location	9
Itoh S. et al. [28]	2022	Japan	64	32	32	69 (44–87)	67 (44–83)	HCC/ICCA/ Metastases/other	Tumor location	9
Jianxi W. et al. [29]	2022	China	162	81	81	NA	NA	HCC	Tumor location, positive/negative staining	9
Liu F. et al. [30]	2023	China	100	50	50	59.16 ± 10.82	56.82 ± 10.41	HCC	Tumor location, positive/negative staining	9
Chen H. et al. [31]	2022	China	108	60	48	56.3 ± 12.1	57.3 ± 9.7	Primary liver cancer	Tumor location, positive/negative staining	7

CRLM- colorectal liver metastases, HCC- hepatocellular carcinoma, ICCA- intrahepatic cholangiocarcinoma, ICG- indocyanine green, NA- not applicable, NETs- neuroendocrine tumors, NOS- Newcastle- Ottawa scale

### Primary outcome

#### R0 resection

**Fig. 2:**
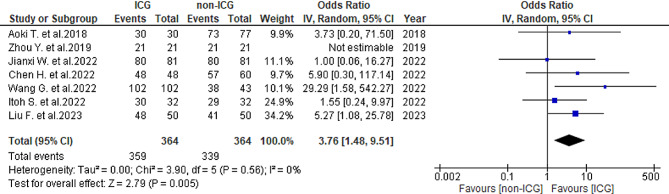
Forest plot displaying R0 resection of the tumors

**Fig. 3:**
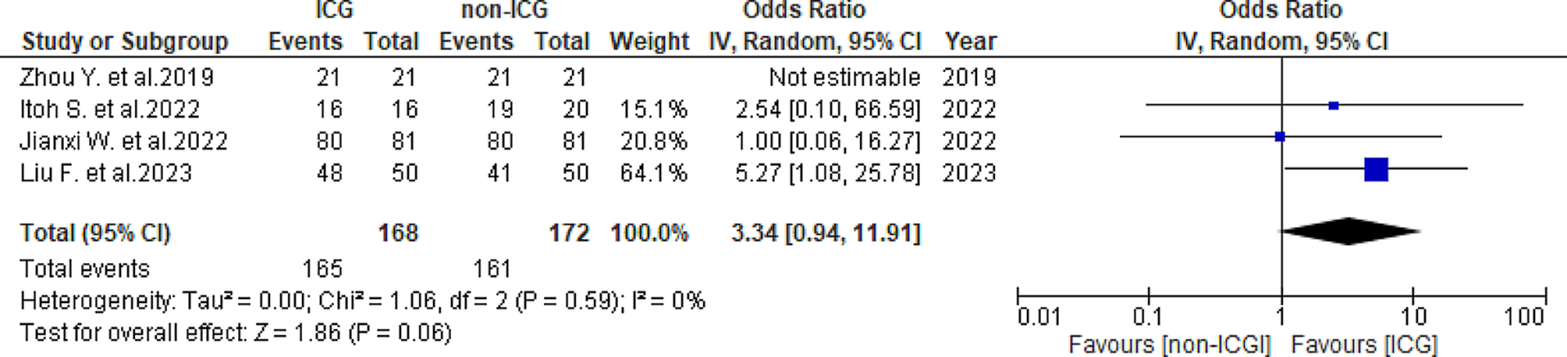
Forest plot displaying R0 resection of HCC subtype

**Fig. 4:**
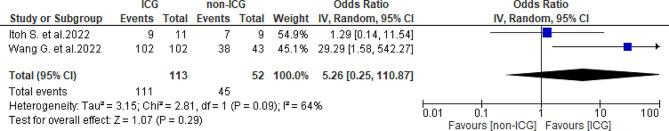
Forest plot displaying R0 resection of liver metastases subtype

In total, seven studies [25–31] reported the R0 resection rate: 98.6% (359/364) in the ICG group and 93.1% (339/364) in the non-ICG group. Heterogeneity was not observed (*I*^2^ = 0%, *P* = 0.56). The ICG group had a significantly higher R0 resection rate (OR = 3.76, 95% CI 1.45–9.51, *P* = 0.005) (Fig. 2). Subgroup analysis was conducted for hepatocellular carcinoma (HCC) and liver metastases. Among the HCC group, four studies [27–30] indicated an R0 resection rate of 98.2% (165/168) in the ICG group and 93.6% (161/172) in the non-ICG group, with no observed heterogeneity (*I*^*2*^ = 0%, *P* = 0.59). The ICG group showed a nonsignificant increase in the R0 resection rate (odds ratio [OR] = 3.34, 95% confidence interval [CI] 0.94–11.91, *P* = 0.06) (Fig. 3). In the liver metastases group, two studies [26, 28] reported an R0 resection rate of 98.2% (111/113) in the ICG group and 86.5% (45/52) in the non-ICG group, with moderate heterogeneity observed (*I*^*2*^ = 64%, *P* = 0.09). The ICG group showed a nonsignificant increase in the R0 resection rate (OR = 5.26, 95% CI 0.25-110.87, *P* = 0.29) (Fig. 4).

### Secondary outcome

#### Margin distance

**Fig. 5:**
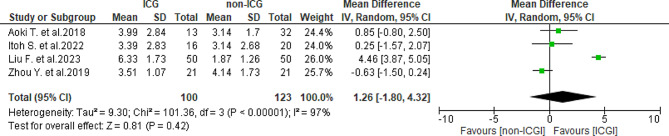
Forest plot displaying margin distance

**Fig. 6:**
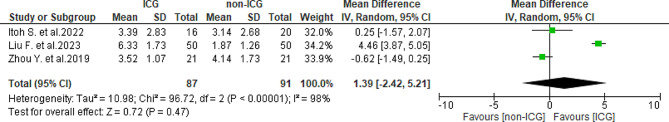
Forest plot displaying margin distance of HCC subtype

A total of four studies [25, 27, 28, 30] reported margin distance, with mean values of 4.96 ± 2.41 millimeters (mm) in the ICG group and 2.79 ± 1.92 mm in the non-ICG group. A significantly large heterogeneity was observed (*I*^2^ = 97%, *P* < 0.001). Overall, no significant differences were found in margin distance between the two groups (WMD = 1.26, 95% CI -1.8-4.32, *P* = 0.42) (Fig. 5). Subgroup analysis for margin distance in HCC, based on three studies [27, 28, 30], showed mean values of 5.11 ± 2.32 mm in the ICG group and 2.67 ± 1.99 mm in the non-ICG group, with significantly large heterogeneity observed (*I*^2^ = 98%, *P* < 0.001). Similarly, no significant differences were found in margin distance between the two groups (WMD = 1.39, 95% CI -2.42-5.21, *P* = 0.47) (Fig. 6).

#### Operative time

**Fig. 7:**
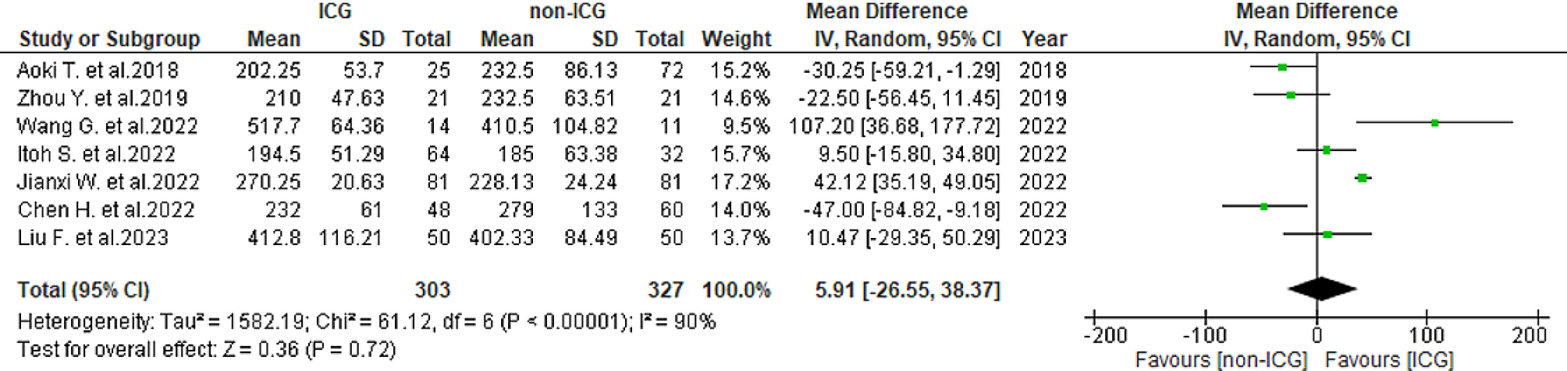
Forest plot displaying operative time

In total, seven studies [25–31] reported the operative time. The mean operative time was 274.5 ± 111 min in the ICG group and 267.3 ± 109.1 min in the non-ICG group. A significantly large heterogeneity was observed (*I*^2^ = 90%, *P* < 0.001). The result of the operative time showed no overall differences in either group (WMD = 5.91, 95% CI -26.55-38.37, *P* = 0.72) (Fig. 7).

#### Intraoperative blood loss

**Fig. 8:**
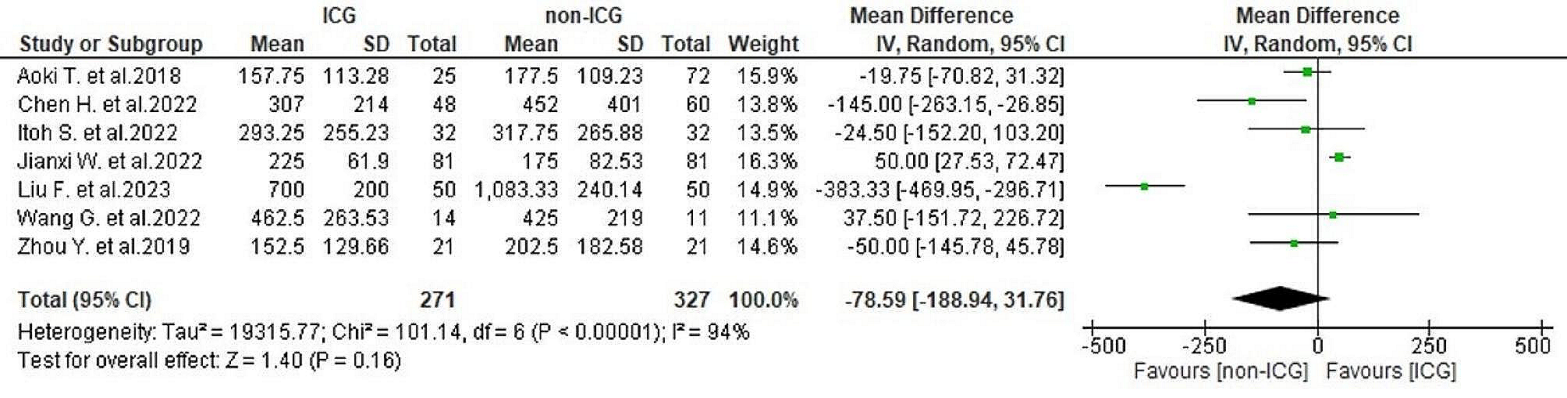
Forest plot displaying intraoperative blood loss

Regarding intraoperative blood loss, data from seven studies [25–31] showed a mean of 319.8 ± 244.3 milliliters (ml) in the ICG group and 389.4 ± 388.6 ml in the non-ICG group. Considerable large heterogeneity was observed (*I*^2^ = 94%, *P* < 0.001). The analysis indicated no significant overall differences in blood loss between the two groups (WMD = -78.59, 95% CI -188.94-31.76, *P* = 0.16) (Fig. 8).

#### Postoperative blood transfusion

**Fig. 9:**
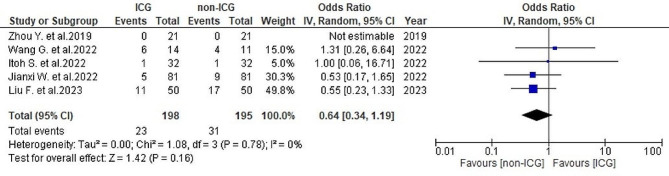
Forest plot displaying postoperative blood transfusion

Postoperative blood transfusion rates were reported in five studies [26–30]: 11.6% (23/198) in the ICG group and 15.9% (31/195) in the non-ICG group. No significant heterogeneity was observed (*I*^2^ = 0%, *P* = 0.78). The analysis showed no substantial overall differences in transfusion rates between both groups (OR = 0.64, 95% CI 0.34–1.19, *P* = 0.16) (Fig. 9).

#### Postoperative length of hospital stay

**Fig. 10:**
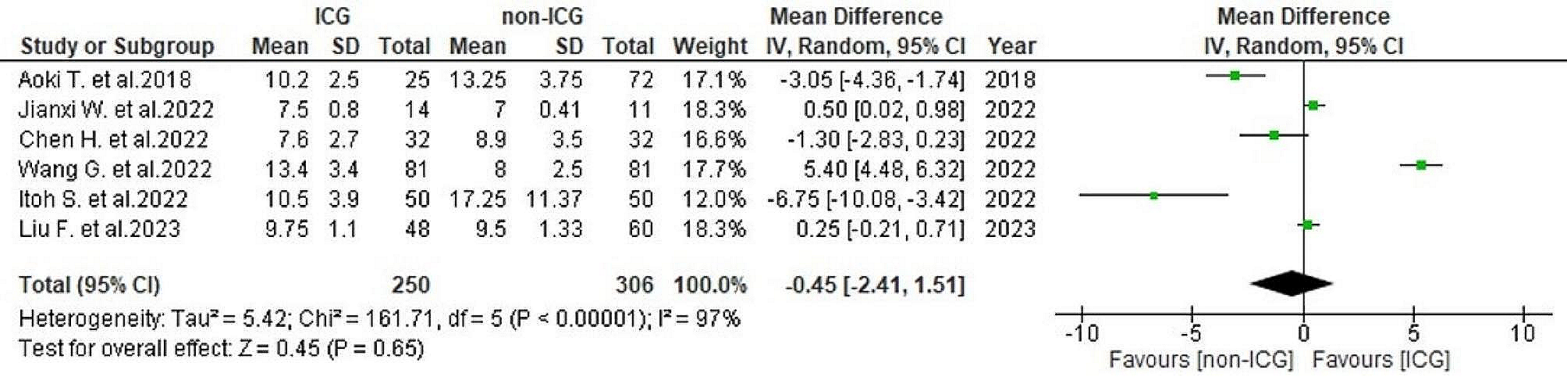
Forest plot displaying postoperative length of hospital stay

In terms of postoperative length of hospital stay, data from six studies [25, 26, 28–31] indicated a mean of 9.8 ± 5.6 days in the ICG group and 10.4 ± 5.5 days in the non-ICG group. Significant heterogeneity was observed (*I*^2^ = 97%, *P* < 0.001). The analysis showed no notable overall differences in hospital stay duration between the two groups (WMD = -0.45, 95% CI -2.41-1.51, *P* = 0.65) (Fig. 10).

#### Postoperative overall complications

**Fig. 11:**
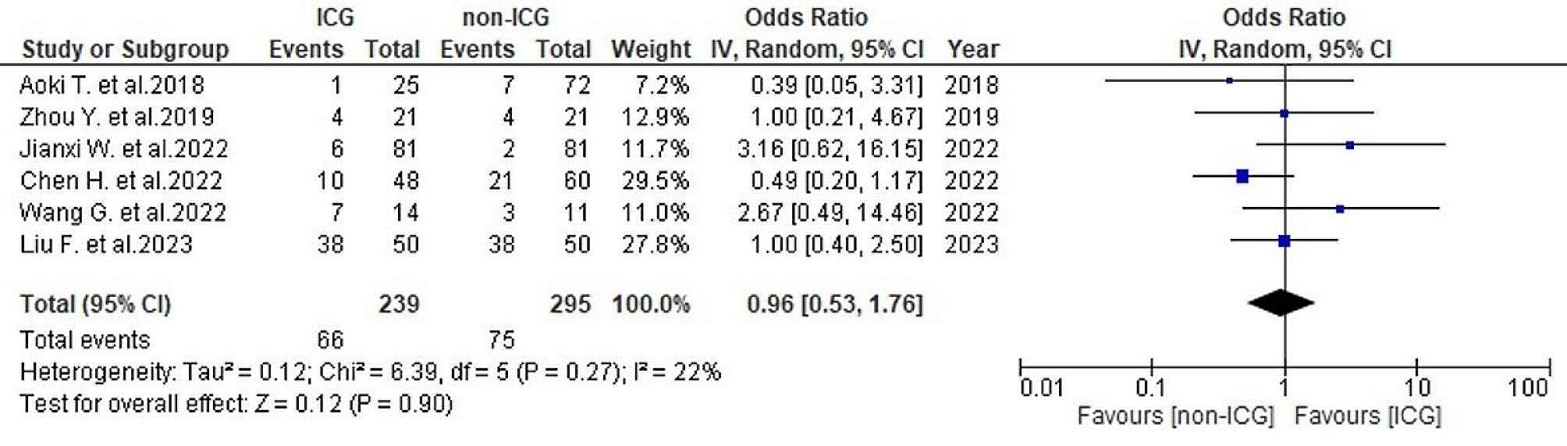
Forest plot displaying postoperative overall complications

A total of six studies [25–27, 29–31] provided data on the postoperative overall complications rate, revealing a rate of 27.6% (66/239) in the ICG group and 25.4% (75/295) in the non-ICG group. Although there was moderate heterogeneity (*I*^2^ = 22%, *P* = 0.27), the analysis indicated no significant disparities between the two groups (OR = 0.96, 95% CI 0.53–1.76, *P* = 0.9) (Fig. 11).

Regarding postoperative bleeding, data from five studies [25–27, 29, 30] showed a rate of 3.1% (6/191) in the ICG group and 2.1% (5/235) in the non-ICG group. In terms of postoperative liver failure, four studies [25–27, 29] reported an incidence of 4.3% (6/141) in the ICG group and 2.7% (5/185) in the non-ICG group. Postoperative bile leakage was reported in five studies [25–27, 29, 30], with rates of 3.1% (6/191) in the ICG group and 3% (7/235) in the non-ICG group. Postoperative abdominal infection data were available from three studies [25, 26, 30], indicating a rate of 2.2% (2/89) in the ICG group and 6% (8/133) in the non-ICG group. Lastly, three studies [25, 26, 30] reported postoperative pleural effusion, with rates of 27% (24/89) in the ICG group and 15.8% (21/133) in the non-ICG group. No significant variability was observed in the mentioned results, and the analysis indicated no significant differences between the two groups (Additional file Figs. S1–S5).

#### Postoperative major and minor complications

**Fig. 12:**
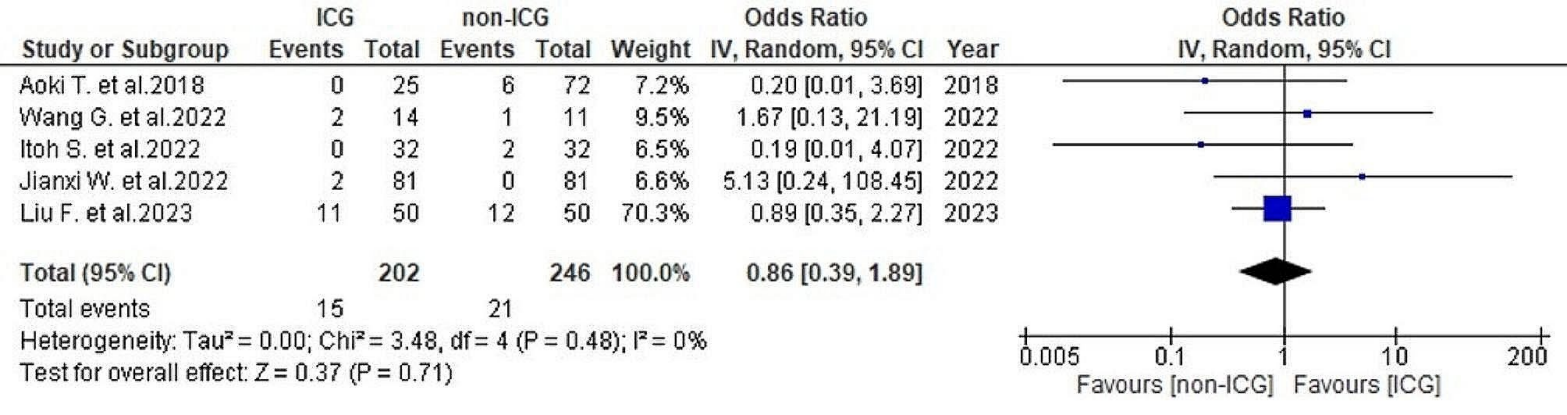
Forest plot displaying postoperative major complication

Across five studies [25, 26, 28–30], the postoperative major complications rate was 7.4% (15/202) in the ICG group and 14.5% (21/246) in the non-ICG group. No heterogeneity was noted (*I*^2^ = 0%, *P* = 0.48). The analysis indicated no significant overall differences between the two groups (OR = 0.86, 95% CI 0.39–1.89, *P* = 0.71) (Fig. 12).

**Fig. 13:**
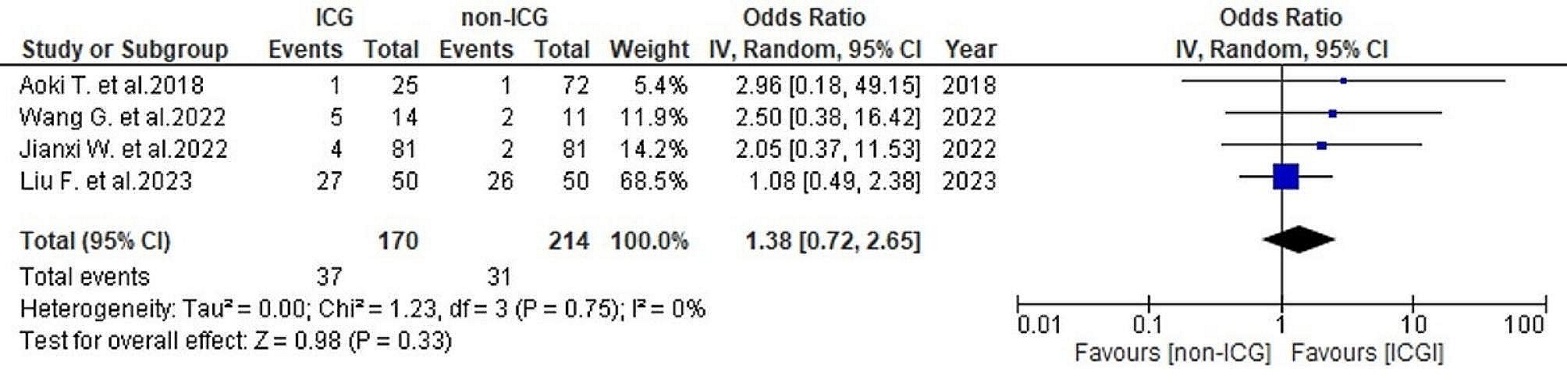
Forest plot displaying postoperative minor complications

In terms of postoperative minor complications, data from four studies [25, 26, 29, 30] revealed a rate of 21.8% (37/170) in the ICG group and 14.5% (31/214) in the non-ICG group. No heterogeneity was detected (*I*^2^ = 0%, *P* = 0.75). The analysis indicated no substantial overall differences between the two groups (OR = 1.38, 95% CI 0.72–2.65, *P* = 0.33) (Fig. 13).

## Discussion

Presently, there exists a range of treatment options for liver cancer, including hepatic resection, ablation, transarterial embolization, chemotherapy, targeted therapy, and radiotherapy. Nevertheless, radical hepatectomy stands as the foremost choice for achieving a curative outcome in liver cancer. The achievement of the R0 resection, indicating the absence of tumor cells in both the microscopic and gross margins, is crucial for minimizing recurrence rates and prolonging survival in all types of liver cancer [32–34]. Consequently, detecting and locating tumors during surgery is the main point to improve the therapeutic effect. Laparoscopic hepatectomy with advanced techniques enables precise resection with smaller incisions, immediate recovery, and no compromise in oncological outcomes. Nonetheless, the complexity of liver anatomy poses challenges in visualizing tumors through laparoscopic means and the absence of tactile sensation of the tumor. IOUS also faces limitations, particularly in cases involving hepatic parenchyma with macronodular cirrhosis and irregular liver surfaces. Additionally, obstacles arise with difficult views and angles due to the position of the trocar, and in achieving real-time imaging during intraparenchymal division [35–37].

ICG fluorescence imaging has recently gained attention and has been used across various fields. In hepatectomy, using ICG-guided laparoscopic hepatectomy techniques enhances precision in resection, identifies tumor boundaries, detects new lesions, provides real-time intraoperative navigation for segment boundaries, and reduces the need for repeated IOUS. Consequently, this leads to wider surgical margins [31, 37, 38]. This method proves to be beneficial even in challenging procedures like laparoscopic right posterior sectionectomy, which involves limited visualization and difficulty in manipulation of laparoscopic devices, as well as in cases of recurrent tumors requiring repeated hepatectomies, achieving a 100% R0 resection rate [27, 39]. However, ICG fluorescence imaging mostly identifies superficial tumors with a depth of 5 to 10 mm and may miss deeper lesions [40–42]. Combining IOUS with ICG fluorescence imaging increases tumor identification sensitivity to 100%, compared to 86% with IOUS alone and 92% with fluorescence imaging alone [40, 43]. Nevertheless, a drawback of this method lies in its relatively high false positive rate, reaching up to 40% for benign nodules like biliary adenofibroma and focal nodular hyperplasia [41, 44]. A report suggested that ICG fluorescence imaging can differentiate between different types of intrahepatic cholangiocarcinoma based on fluorescence patterns [45]. Additionally, a significant correlation was observed between ICG fluorescence patterns and grade of differentiation of HCC, with a uniform fluorescence pattern predominantly found in well-differentiated HCC, while partially and rim-type fluorescence patterns were observed in moderately and poorly differentiated HCC, respectively [46].

The efficacy of ICG fluorescence imaging in influencing oncological outcomes remains uncertain. All prior studies have been retrospective cohort analyses, with few providing long-term outcomes. Previous research indicated that overall survival (OS) and recurrence-free survival (RFS) did not exhibit significant differences [29, 47]. Among the studies included in this meta-analysis, only two provided data on the long-term follow-up of the HCC patients. One study reported significantly improved RFS in the ICG group [30], while OS did not differ between the two groups in both studies [29, 30]. The first study reported a significantly improved 6-month and 18-month RFS rate in the ICG group, which were 90% and 80%, respectively, compared to 82% and 66% in the non-ICG group. Additionally, the 6-month and 18-month OS rates in the ICG group were 98% and 88%, respectively, compared to 98% and 84% in the non-ICG group, with no significant difference observed. The follow-up period in this study was less than three years [30]. Another study demonstrated that the 1-year, 2-year, 3-year, and 4-year RFS rates in the ICG group were 86.5%, 69.7%, 58.7%, and 44%, respectively, compared to 81.6%, 75.6%, 72%, and 67.9% in the non-ICG group. Likewise, the 1-year, 2-year, 3-year, and 4-year OS rates in the ICG group were 96.1%, 92.2%, 89.6%, and 80.6%, respectively, compared to 93.5%, 90.8%, 80.9%, and 77% in the non-ICG group, with no significant difference observed [29].

This systematic review and meta-analysis include all available recent studies. This meta-analysis of seven high-quality studies with 598 patients indicates that ICG fluorescence-assisted laparoscopic hepatectomy significantly enhances the rate of achieving R0 resections, without extending operative time, increasing blood loss, prolonging hospital stays, or leading to additional complications. Notably, one study noted two patients in the ICG group with positive margins, attributed to infrequent conversions to fluorescence imaging during the operation and the challenging location of the tumor in segments 7 and 8 [28]. The achievement of negative margins and optimal resection margins holds significance in improving OS and RFS for HCC. Numerous studies have demonstrated that a wide margin exceeding one centimeter establishes superior outcomes compared to a narrow margin [34, 48]. In our subgroup analysis specifically targeting R0 resection in HCC, we observed a trend towards wider margins associated with higher OR, although the results were not statistically significant. Additionally, the margin analysis conducted in this study did not reveal statistically significant differences. Therefore, further investigation is necessary to verify this hypothesis.

Heterogeneity was noted in terms of operative time, intraoperative blood loss, and postoperative hospitalization. To address this, we excluded certain studies that could potentially contribute to this variability. However, even after this adjustment, significant heterogeneity persisted, and there was no significant difference in the outcomes. Upon analyzing the weighted mean differences in continuous variables compared to standardized mean differences revealed the same results (Additional file Figs. S6–S10). Minimally invasive liver surgery aims to reduce the length of hospital stay. Our findings indicated that the mean hospital stay was 9.8 ± 5.6 days in the ICG group and 10.4 ± 5.5 days in the non-ICG group, which seems comparatively long. A previous study comparing hospital stays between open and laparoscopic surgery reported a mean stay of 11.3 days for the open group and 6.2 days for the laparoscopic group [6]. The median hospital stay was 9 to 10 days for open surgery and 7 to 8 days for laparoscopic surgery [7, 49]. Prior studies on ICG-assisted open hepatectomy reported a mean hospital stay of 10.4 to 25.75 days in the ICG group, compared to 13.4 to 18.2 days in the conventional group [47, 50, 51]. Therefore, the hospital stay for laparoscopic hepatectomy in our review appears shorter than that for open ICG-guided surgery. Regarding the conversion rate, an aspect of concern for minimally invasive surgeons, only one study reported that one patient (2%) in the ICG group had a significantly lower rate of conversion to open surgery compared to seven patients (14%) in the non-ICG group [30].

Robot-assisted hepatectomy is increasingly gaining popularity. ICG-guided robotic surgery has proven to be beneficial and shown promising results, including a resection margin of 10 mm [52], a 100% R0 resection rate, and the detection of previously missed lesions [53]. Although many articles have been published, most are case series with sample sizes of less than 50 [54–56]. Therefore, prospective studies with larger sample sizes are needed for a more comprehensive analysis.

Nevertheless, some limitations of the study need to be addressed. All included studies were retrospective cohort studies, which carry the risk of missing data, and the possibility that significant biases may have occurred in the selection of controls. This analysis may be subject to bias due to the small sample size in some studies, as ICG-guided laparoscopic hepatectomy represents a relatively new approach. Given the numerous techniques available for applying ICG to assist in surgical procedures, the methods of ICG application varied among the studies included in this analysis. These methods include tumor staining as well as positive or negative staining to distinguish the transection line. The lack of a standardized ICG administration protocol for this modality introduces the potential for bias. Moreover, all studies were conducted in Asia and there were also discrepancies in the types of liver cancer included in the study. Heterogeneity was noted in results related to operative time, intraoperative blood loss, and postoperative hospitalization. As the studies reported since 2018, data on long-term follow-up is limited. We recommend further research employing randomized controlled trials with standardized protocols, larger sample sizes, and extended follow-up durations to assess long-term RFS and OS rates.

## Conclusions

The utilization of ICG-guided laparoscopic hepatectomy proves helpful in detecting tumors and visualizing margins throughout the surgical procedure. ICG-guided laparoscopic hepatectomy helps identify tumors and see the margin during surgery. Given the challenge of palpating the tumor during surgery, this approach can assist with tumor detection and provide real-time segmentation of the liver during the transection. This study concludes that the ICG group has a significantly superior R0 resection rate which might lead to promising oncologic outcomes without a concurrent rise in complications. Subsequent research should focus on the extended follow-up of OS and RFS rates to validate the applicability of this technique.

### Electronic supplementary material

Below is the link to the electronic supplementary material.

Supplementary Material 1

## Data Availability

The datasets used and analyzed during the current study are available from the corresponding author on reasonable request.
