# Supplementary material for: Surgical margin status outcome of intraoperative indocyanine green fluorescence-guided laparoscopic hepatectomy in liver malignancy: a systematic review and meta-analysis
Source: BMC Surg. 2024 Jun 12;24:181. doi: 10.1186/s12893-024-02469-1 (PMC11167816; doi:10.1186/s12893-024-02469-1)

**Supplementary material**

**Table S****1. Quality assessment of the included studies using the Newcastle-Ottawa Quality Assessment Scale**

| Authors | Selection  (From total of ●●●●) | Comparability  (From total of ●●) | Outcome  (From total of ●●●) |
| --- | --- | --- | --- |
| Aoki T. et al. 2018 | ●●●● | ●● | ●●● |
| Wang G. et al. 2022 | ●●●● | ● | ●●● |
| Zhou Y. et al. 2019 | ●●●● | ●● | ●●● |
| Itoh S. et al. 2022 | ●●●● | ●● | ●●● |
| Jianxi W. et al. 2022 | ●●●● | ●● | ●●● |
| Liu F. et al. 2023 | ●●●● | ●● | ●●● |
| Chen H. et al. 2022 | ●●● | ● | ●●● |

**Figure S1. Forest plot displaying postoperative bleeding**

**
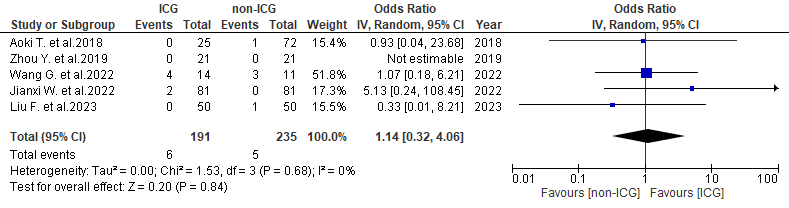
**

**Figure S2. Forest plot displaying postoperative liver failure**

**
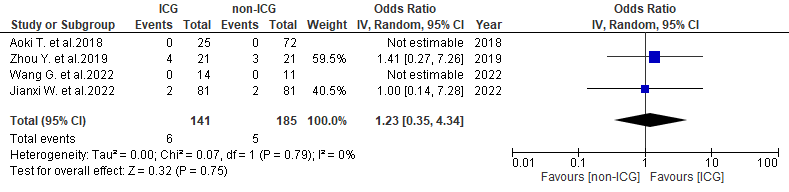
**

**Figure S3. Forest plot displaying postoperative bile leakage**

**
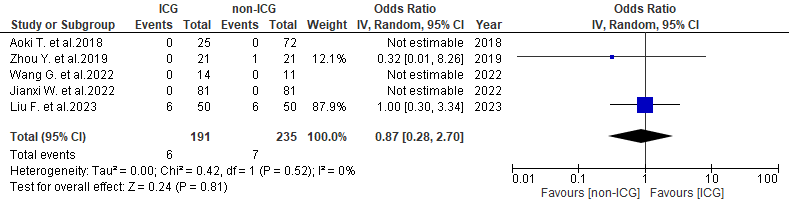
**

**Figure S4. Forest plot displaying postoperative abdominal infection**

**
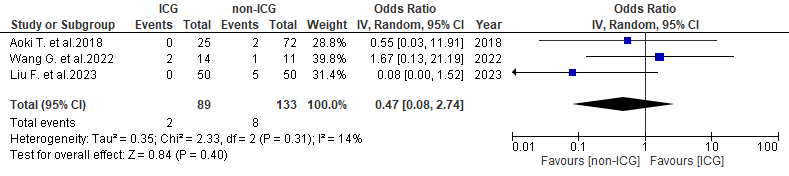
**

**Figure S5. Forest plot displaying postoperative pleural effusion**

**
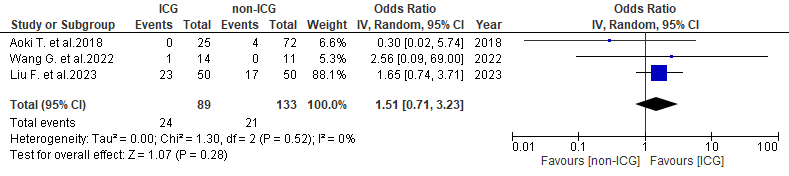
**

**Figure S6. Forest plot displaying margin distance with standardized mean difference**

**
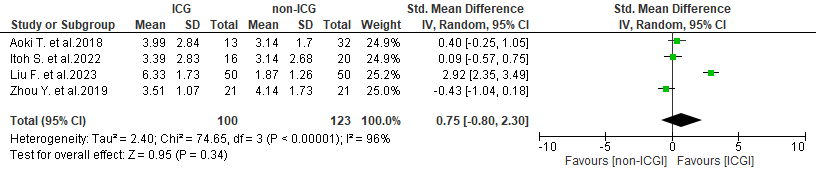
**

**Figure S7. Forest plot displaying margin distance of HCC subtype with standardized mean difference**

**
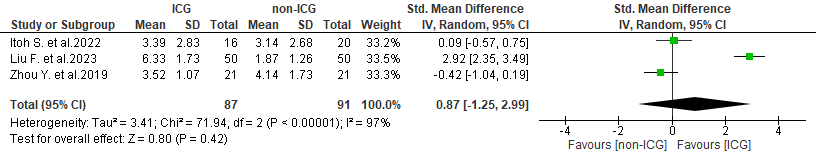
**

**Figure S8. Forest plot displaying operative time with standardized mean difference
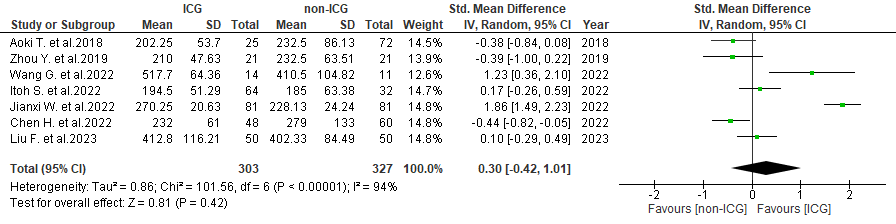
**

**Figure S9. Forest plot displaying intraoperative blood loss with standardized mean difference**

**
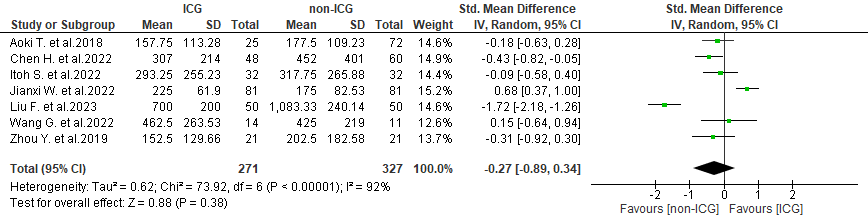
**

**Figure S10. Forest plot displaying postoperative length of hospital stay with standardized mean difference
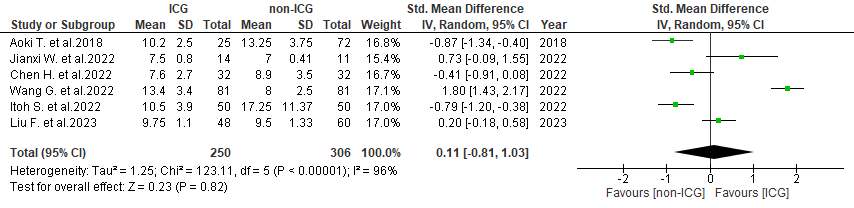
**

**Figure S11. Funnel plot of the included studies in the meta-analysis of** **R0 resection of the tumors**


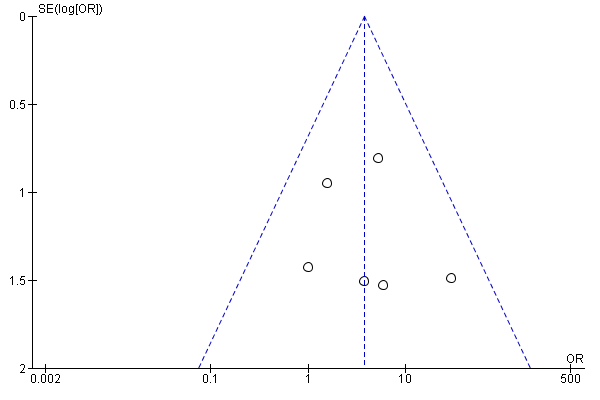


**Figure S12. Funnel plot of the included studies in the meta-analysis of** **R0 resection of HCC subtype**

**
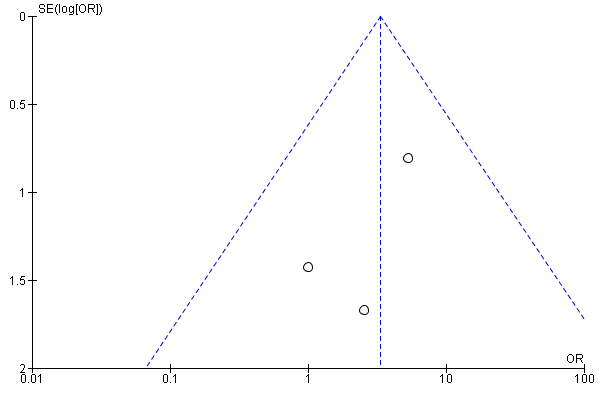
**

**Figure S13. Funnel plot of the included studies in the meta-analysis of** **R0 resection of liver metastases subtype**

**
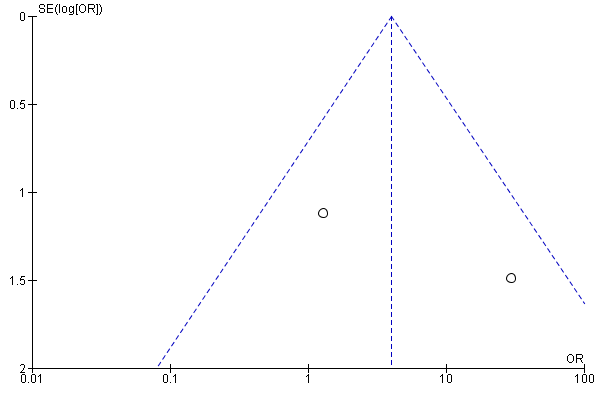
**

**Figure S14. Funnel plot of the included studies in the meta-analysis of** **margin distance**

**
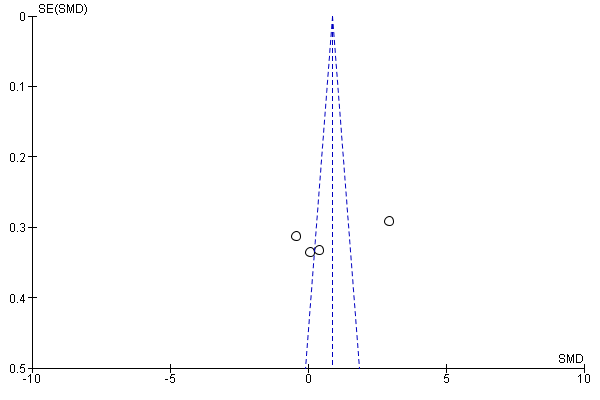
**

**Figure S15. Funnel plot of the included studies in the meta-analysis of** **margin distance of HCC subtype**

**
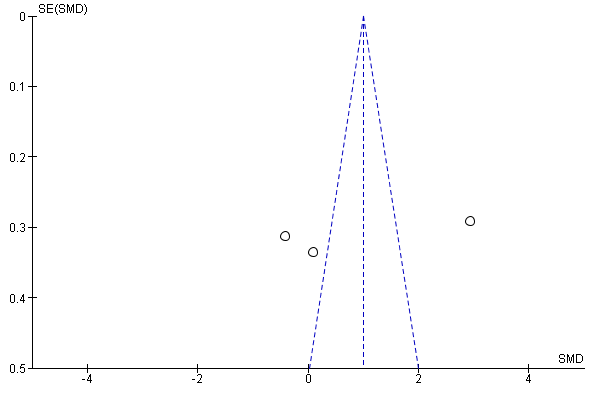
**

**Figure S16. Funnel plot of the included studies in the meta-analysis of operative time**

**
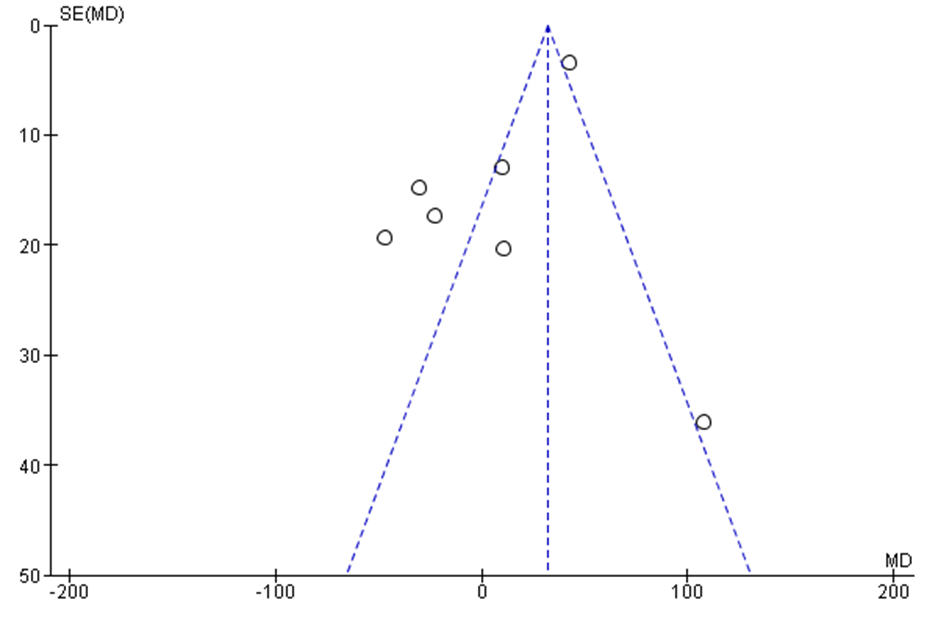
**

**Figure S17. Funnel plot of the included studies in the meta-analysis of** **intraoperative blood loss**


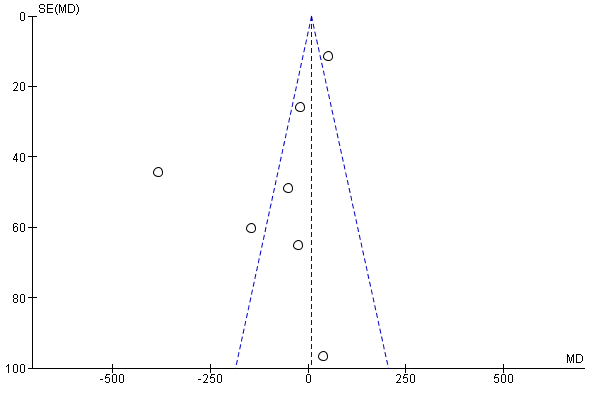


**Figure S18. Funnel plot of the included studies in the meta-analysis of postoperative blood transfusion**


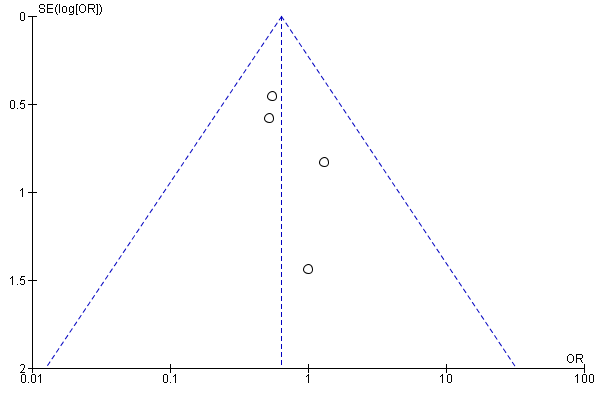


**Figure S19. Funnel plot of the included studies in the meta-analysis of postoperative length of hospital stay**

**
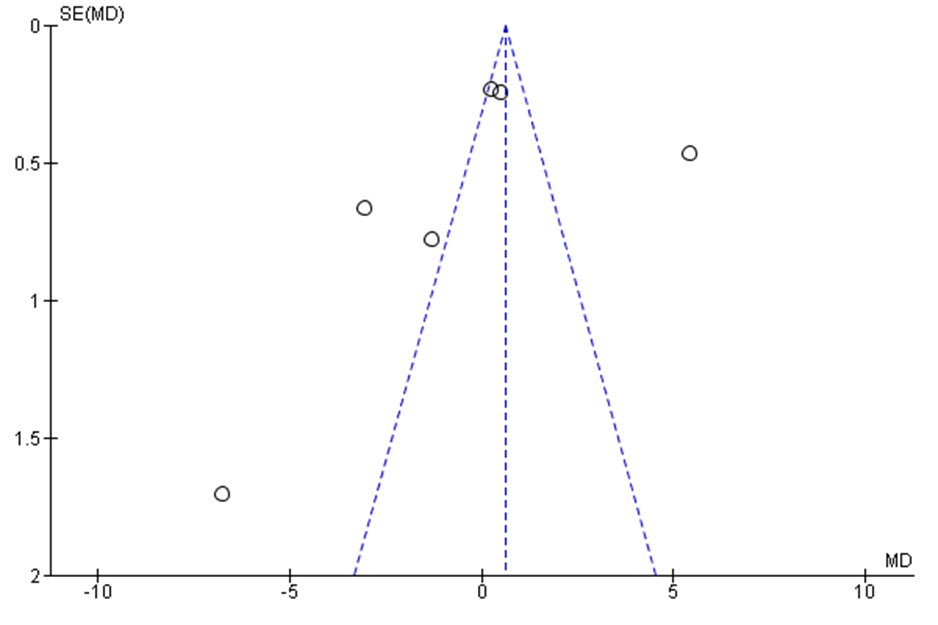
**

**Figure S20. Funnel plot of the included studies in the meta-analysis of** **postoperative overall complications**


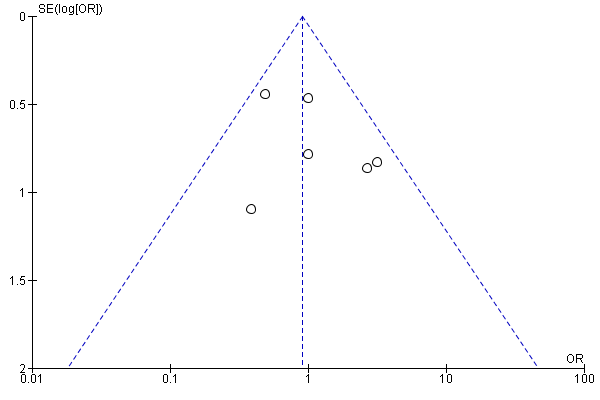


**Figure S21. Funnel plot of the included studies in the meta-analysis of** **postoperative major complications**


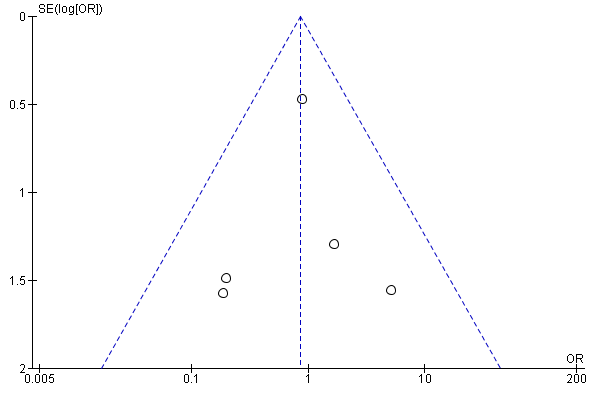


**Figure S22. Funnel plot of the included studies in the meta-analysis of** **postoperative minor complications**


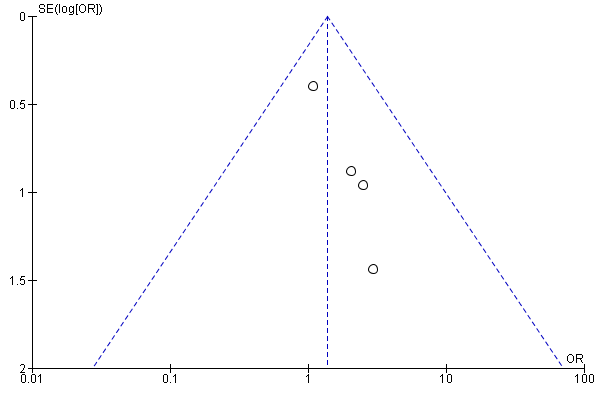


**Figure S23. Funnel plot of the included studies in the meta-analysis of** **postoperative bleeding**


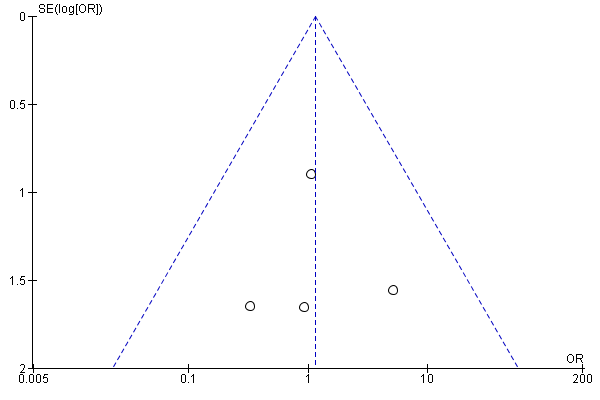


**Figure S24. Funnel plot of the included studies in the meta-analysis of** **postoperative liver failure**


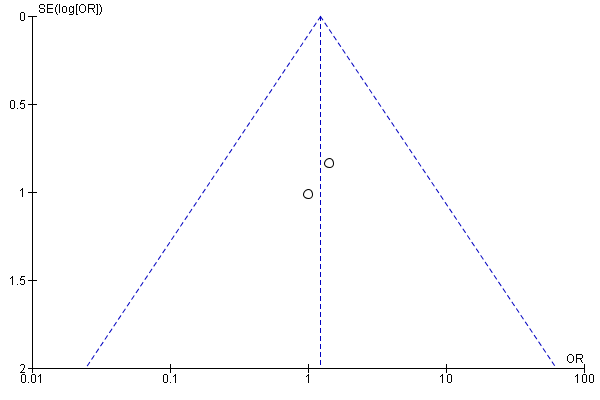


**Figure S25. Funnel plot of the included studies in the meta-analysis of** **postoperative bile leakage**


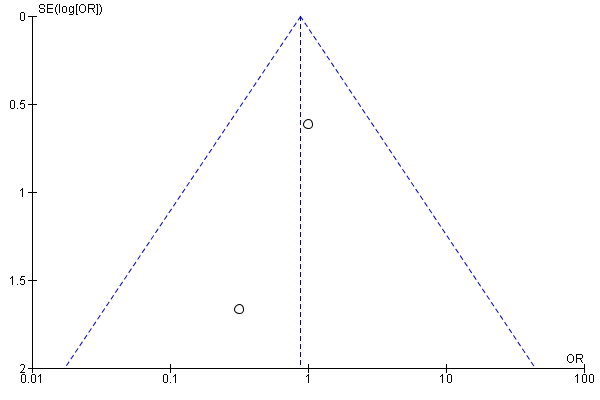


**Figure S26. Funnel plot of the included studies in the meta-analysis of** **postoperative abdominal infection**


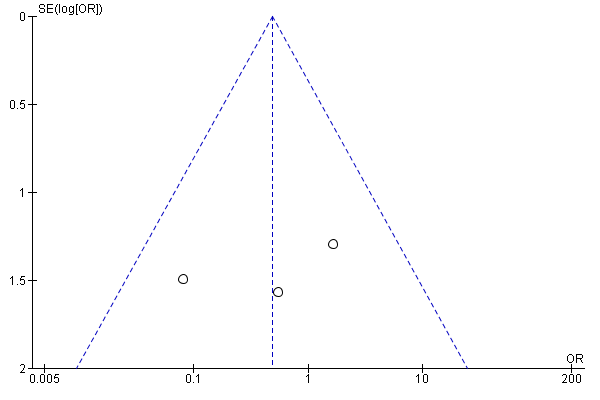


**Figure S27. Funnel plot of the included studies in the meta-analysis of** **postoperative pleural effusion**


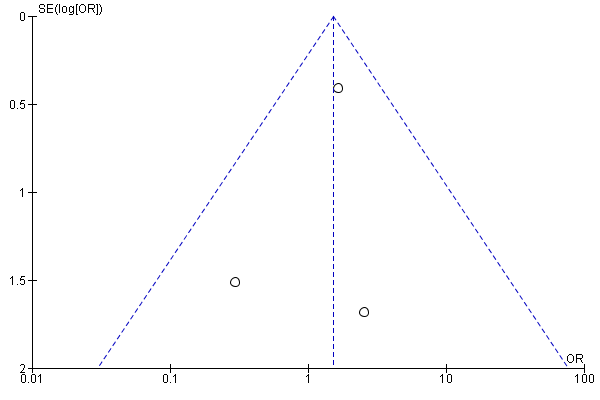

Supplement: Supplementary file 1 — Supplementary Material 1 [file 12893_2024_2469_MOESM1_ESM.docx]
